# Motor Cortex Representation of the Upper-Limb in Individuals Born without a Hand

**DOI:** 10.1371/journal.pone.0018100

**Published:** 2011-04-08

**Authors:** Karen T. Reilly, Angela Sirigu

**Affiliations:** 1 CNRS, Cognitive Neuroscience Center, UMR 5229, Bron, France; 2 University Lyon 1, Villeurbanne, France; The University of Western Ontario, Canada

## Abstract

The body schema is an action-related representation of the body that arises from activity in a network of multiple brain areas. While it was initially thought that the body schema developed with experience, the existence of phantom limbs in individuals born without a limb (amelics) led to the suggestion that it was innate. The problem with this idea, however, is that the vast majority of amelics *do not* report the presence of a phantom limb. Transcranial magnetic stimulation (TMS) applied over the primary motor cortex (M1) of traumatic amputees can evoke movement sensations in the phantom, suggesting that traumatic amputation does not delete movement representations of the missing hand. Given this, we asked whether the absence of a phantom limb in the majority of amelics means that the motor cortex does not contain a cortical representation of the missing limb, or whether it is present but has been deactivated by the lack of sensorimotor experience. In four upper-limb amelic subjects we directly stimulated the arm/hand region of M1 to see 1) whether we could evoke phantom sensations, and 2) whether muscle representations in the two cortices were organised asymmetrically. TMS applied over the motor cortex contralateral to the missing limb evoked contractions in stump muscles but did not evoke phantom movement sensations. The location and extent of muscle maps varied between hemispheres but did not reveal any systematic asymmetries. In contrast, forearm muscle thresholds were always higher for the missing limb side. We suggest that phantom movement sensations reported by some upper limb amelics are mostly driven by vision and not by the persistence of motor commands to the missing limb within the sensorimotor cortex. We propose that prewired movement representations of a limb need the experience of movement to be expressed within the primary motor cortex.

## Introduction

In order to control actions of their bodies humans need constant information about the state and position of body parts. For this information to be useful in the planning and execution of actions it needs to be mapped onto an internal representation of the body. This action-related representation is generally referred to as the body schema; a type of sensorimotor representation of the body [e.g. 1], [2]. While there is widespread agreement that the body schema refers to a range of different sensorimotor representations of the body [see 3 for a discussion], there is still an ongoing debate over whether this body schema is innate or acquired [4], [5]. For many years the dominant view was that the body schema required experience and was the result of developmental processes [e.g. 6], [7]. By the end of the 1960s, however, the discovery of phantom limbs in a few individuals born without the limb (amelics) was cited as evidence for the existence of an innate body schema representation in the brain [e.g. 8]. Melzack and colleagues did a mail-out survey study and found that 18% of respondents born without a limb reported the presence of a phantom [9], but a similar study published a year later reported an incidence of only 8% [10]. Despite the low incidence of phantom sensations in amelic subjects compared with subjects who are amputated later in life, some cases are well documented, and are held up as evidence in favour of the existence of a hard-wired, genetically predetermined body schema [9], [11], [12], [13]. Others, however, argue that such phantom limbs do not prove the existence of an innate body representation, but could instead arise from learning through observation [see 14].

The presence of phantoms in amelic individuals does appear to be a strong argument in favour of the existence of some form of innate body schema. But their presence in some individuals raises the question of why between 80 and 90% of individuals born with congenital absence of a limb *do not* possess a phantom limb. One possibility is that these people are born with a body schema that represents a whole human body but that the visual information signalling the absence of the body part overrides the innate body schema. Another option is that instead of being deleted, the body part's representation is simply latent or temporarily inhibited. As such, the person reports no sensations related to the missing limb not because their body schema no longer contains a representation of the missing limb, but because its representation has been deactivated or inhibited and thereby rendered inaccessible to conscious (or even unconscious) experience. Indeed, there are at least three documented cases of individuals born without a limb who, following minor surgery or injury, experienced a phantom for the first time as adults [15]. This suggests that people born without a limb who do not have a phantom could have an intact (whole-body) body schema with parts of the body schema remaining latent throughout life.

Transcranial magnetic stimulation applied over the primary motor cortex (M1) of traumatic amputees who report the presence of a phantom limb can evoke the sensation of movement in the phantom [16], [17], [18], [19]. This, together with the fact that amputees report being able to move their phantom voluntarily [20], [21], [22], suggests that their body schema maintains a motor representation of the missing hand and that this representation can be activated by stimulating the motor cortex [23]. Importantly, TMS can evoke movements of the phantom limb that traumatic amputees cannot produce voluntarily [19], suggesting that the inability to make a particular phantom movement voluntarily does not mean that the movement representation no longer exists. Following this idea, we asked whether the absence of phantom sensations (as is the case in the majority of individuals born without a limb) means that a motor representation of the missing limb does not exist, or whether it is present in the primary motor cortex but cannot be accessed voluntarily.

We also asked whether the absence of phantom sensations was related to the organisation of the sensorimotor cortex. Nearly 80% of traumatic upper-limb amputees report phantom sensations [24], and numerous studies examining the organisation of the motor cortices in these patients report between-hemisphere differences in the location of body part or stump muscle representations [25], [26], [27], [28], [29], [30], [31], and in stump muscle resting motor thresholds [17], [19], [25], [30], [32], [33]. Since these parameters are relatively symmetric in normal populations [e.g. 34], [35], the presence of asymmetries between the two motor cortices might be related to the persistence of a motor representation of the missing limb as well as its reorganisation following amputation.

While some studies examining the body's representation within the sensorimotor cortex of individuals born without a limb do exist they do not paint a clear picture of how congenital absence of one limb affects the symmetry of sensorimotor representations. Two studies examining tactile-evoked sensory responses reported that tactile stimulation of the mouth resulted in symmetric activation of the two hemispheres [36], [37], but the results from the motor domain differ. Funk and colleagues [38] used fMRI to assess the symmetry of the tongue's motor representation and found that horizontal tongue movements were represented more medially within the sensorimotor cortex contralateral to the missing limb, a finding also reported in some traumatic amputees [39], [40]. Kew and colleagues [41] examined motor representations of the upper-limb in both congenital and traumatic amputees using positron emission tomography (PET) and TMS. They found that when congenital amputees made paced shoulder movements with their affected arm the blood flow increase in the sensorimotor cortex occurred over a wider area but was not significantly more intense than that recorded when they moved their intact arm. In traumatic amputees, however, movements of the amputated side were associated with increased blood flow both in terms of intensity and area. When they performed TMS on these subjects they found that in the three traumatic amputees responses were evoked from more scalp sites for the stump muscle than for the intact muscle, but that this was not the case for the three congenital amputees.

Overall these studies show that the tongue representation is symmetric in the sensory cortex but asymmetric in the motor cortex, and the upper-limb motor representation is asymmetric when subjects make voluntary movements (PET study), but symmetric (as measured by muscle map area) when muscles are tested at rest (TMS study). While these studies give us some information about the symmetry of sensorimotor representations in amelics, none of them explicitly set out to investigate the symmetry of upper-limb muscle representations, and it is particularly unclear whether their organisation resembles the *re*-organisation observed after traumatic amputation.

In this study we investigated the cortical representation of hand movements in subjects born without their upper limb below the elbow. First we determined whether phantom sensations or movements could be elicited by direct stimulation of the arm/hand region in M1. Second, we examined between-hemisphere differences in the location of upper-limb muscle representations and in their motor thresholds. To do this we used image-guided TMS to systematically map the biceps brachii (BB) and flexor digitorum superficialis (FDS) on the intact and missing limb sides. In order to localise the “probable” hand area in the hemisphere contralateral to the missing limb we also mapped an intrinsic hand muscle on the intact side, the first dorsal interosseous (FDI).

## Materials and Methods

### Ethics Statement

The Ethical Committee at the Centre Léon Berard, Lyon, France approved the experimental procedure, which conformed to the Declaration of Helsinki, and all participants included in the study provided written consent.

### Subjects

Four females born with part of their forearm and no hand, and aged between 25 and 38 participated in the study. Two subjects (S1 and S2) had agenesis of the left forearm below the elbow. The other two subjects (S3 and S4) experienced an in utero amputation of the right forearm below the elbow. None of the subjects had ever experienced phantom sensations. Table 1 details the characteristics of each subject.

**Table 1:** Subject Characteristics.

Subject	Age	Missing limb Side	Stump length	Prosthesis type and usage frequency	Cause
S1	25	Left	8 cm	Cosmetic, everyday	agenesis
S2	30	Left	8 cm	Cosmetic, everyday	agenesis
S3	35	Right	10 cm	Cosmetic, everyday	in utero amputation
S4	38	Right	10 cm	None	in utero amputation

### Sensations evoked during TMS Mapping

In our previous study with traumatic upper-limb amputees motor cortex stimulation evoked sensations of movement within the phantom limb [19]. Thus, before each TMS session we explained to the subjects that we were going to stimulate their motor cortex, that this would evoke twitches in their muscles, and that it might also evoke various sensations in their stump and arm. We emphasised the possibility that they could experience the temporary presence of their missing hand and/or movements within this missing hand. We asked subjects to pay careful attention to these types of sensations and, after stimulation at each site, to verbally report any sensations that they experienced.

### TMS Mapping Procedure

Surface EMG was recorded from three muscles on the intact side; biceps brachii (BB), flexor digitorum superficialis (FDS), and first dorsal interrosseus (FDI), and two muscles on the missing limb side; BB and FDS. While we have called the muscle recorded from the stump FDS, it is nearly impossible to be sure which muscles remain in the stump and it is likely that our surface recordings picked up activity from the group of forearm flexors. To record from the intact side we placed the electrodes the same distance from the elbow as those placed on the missing limb side, so we maximized the chance that we recorded from the same muscle group on both sides of the body. Electrodes, 10 mm in diameter (VerMed, Bellows Falls, VT), were placed in a bipolar configuration over each muscle such that signal from the target muscle was optimized. EMG activity was amplified by a factor of 5,000–20,000 and band pass filtered to produce a signal that fell within a ±5 V range (Neurolog Instruments; Digitimer Ltd). Spike2 software and a Power 1401 interface (Cambridge Electronic Design, Cambridge, UK) were used to collect surface EMG data at 1000 Hz.

TMS was applied using a Magstim 200 (Magstim, Dyfed, UK) stimulator connected to a 70-mm figure-of-eight coil. The stimulation intensity for delivery of TMS was different for each subject and each muscle, and was based upon the muscle's at-rest activation threshold. For each muscle we first identified the optimal location for TMS-activation by placing the coil near the estimated hand area of the motor cortex, stimulating at a suprathreshold intensity, then moving the coil in 10 mm steps in order to identify the stimulation site that evoked the largest MEPs. Once this site was identified the resting motor threshold was determined as the minimal intensity of stimulation that produced MEPs larger than 50 µV in 50% of the stimulations delivered to this site.

To facilitate the mapping, subjects wore a tight cap with a grid consisting of a set of points placed 10 mm apart. We were able to obtain a precise estimate of the stimulation site by localizing the TMS coil relative to the brain surface. This was achieved by first acquiring an anatomical magnetic resonance image (MRI) of the brain of each subject. We then used this image to achieve real-time guidance of the position of the stimulating coil relative to the brain surface [42]. To do this we co-registered the subject's MRI with the actual position of her head by placing a Polhemus receiver on the forehead and then measuring the 3-D location of 200 points on the scalp with an electromagnetic position sensor (Polhemus Isotrack II®).

During the construction of the TMS maps stimulation intensity was set at 110% of the subject's resting motor threshold. Maps were constructed by stimulating each grid locus with one stimulation train (six pulses with random inter-pulse intervals of between 3 and 5 seconds). The number and extent of cortical sites stimulated differed for each subject as we continued to stimulate at various grid loci until sites at the boundary of the stimulated area no longer evoked MEPs.

A custom-made program was used to measure MEP latencies and peak-to-peak amplitudes from the EMG recordings. For each muscle we calculated the mean MEP amplitude at each stimulated point and then projected these values onto the brain in order to create separate cortical representation maps of each muscle. We then computed the centres of gravity (COG) of the maps [42] and calculated the medio-lateral distance of the CoGs from the midline. Map area was calculated as the number of active sites (i.e. sites with a mean MEP amplitude ≥0.05 mV).

## Results

### Sensations evoked during TMS Mapping

Stimulation over the hemisphere contralateral to the missing limb was applied at 110% of the resting motor threshold of BB and FDS. Thus, stimulation intensities differed for each subject and for each muscle and are shown in Table 2. Stimulation over the arm/hand area in the hemisphere contralateral to the missing limb *never* induced the sensation that the missing limb was present nor sensations of movement in the missing limb (i.e. stimulation never resulted in the manifestation of a phantom limb). The majority of the stimulation sites were anterior to the central sulcus (predominantly over motor cortical areas), but some posterior sites were also stimulated. Thus, in all four subjects, stimulation of primary motor, premotor, and primary sensory cortices did not evoke phantom sensations. All subjects felt stimulation-induced muscle contractions in both their amelic and intact arms. One subject also reported that stimulation evoked a tingling sensation within the stump at 20% of the stimulation sites on the hemisphere contralateral to the stump, but this same type of tingling sensation was also reported by her at 5% of the stimulation sites on the opposite hemisphere. Sites that evoked tingling sensations were found predominantly over the motor and premotor cortices for both hemispheres. Another subject had a similar tingling sensation in the stump but only at two of the 43 stimulation sites contralateral to the stump. She did not report any sensations during stimulation contralateral to the intact side. The other two subjects reported no stimulation-related sensory sensations in the stump or their intact hand and forearm.

**Table 2:** Stimulation Intensities (% of maximal stimulator output) used to map each muscle.

	Missing Limb Side		Intact Side	
Subject	Muscle	Stimulation Intensity	Muscle	Stimulation Intensity
S1	BB	77	BB	78
	FDS	71	FDS	62
			FDI	64
S2	BB	78	BB	78
	FDS	81	FDS	55
			FDI	57
S3	BB	56	BB	83
	FDS	64	FDS	56
			FDI	50
S4	BB	67	BB	81
	FDS	64	FDS	54
			FDI	53

### TMS Mapping Results

**Figure 1:**
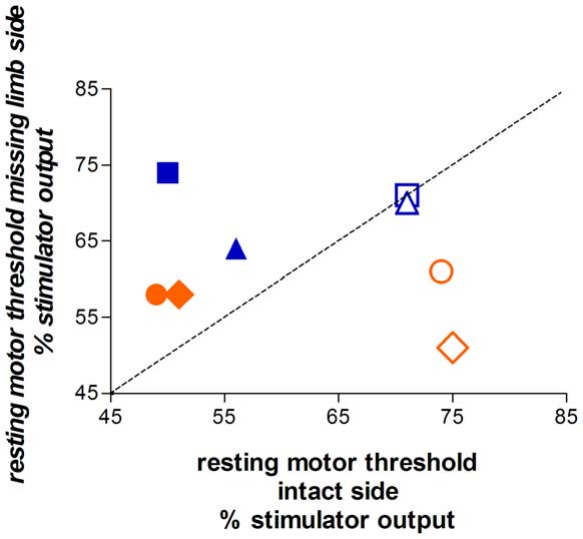
Resting motor thresholds for BB (open symbols) and FDS (filled symbols) on the missing and intact limb sides of each subject (blue = agenesics; orange = in utero amputees). For all four subjects the resting motor thresholds for the stump muscle FDS were higher on the missing limb side than the intact side. The BB thresholds showed the opposite pattern for two subjects and were equivalent on both sides for the other two subjects. (triangle = S1, square = S2; circle = S3, diamond = S4).

In all four subjects, the threshold for activation of FDS on the missing limb side was always *higher* (by an average of 12%) than on the intact side (Figure 1). This contrasts with results from traumatic **above-elbow** adult amputees, in whom the threshold for activation of the muscle immediately proximal to the amputation is always *lower* on the amputated than the intact side, with the mean difference ranging from 10 to 17% [17], [19], [25], [30], [33]. The difference between our results and those typically reported for traumatic amputees might arise from the fact that all four subjects were missing their limb below the elbow. Indeed, data from traumatic **below-elbow** adult amputees suggest that changes might be less systematic than those observed following above-elbow amputation. In two below elbow amputees Kew and colleagues [41] reported no threshold difference for a forearm wrist muscle on the intact and amputated sides. In addition, recent pilot data from three below-elbow amputees show that motor thresholds for the forearm stump muscles were either the same, higher, or lower than those of homologous muscles on the intact side (C. Mercier, personal communication).

While FDS thresholds were always higher on the missing limb side, biceps thresholds on the missing limb side were either equal to (S1 and S2) or lower (S3 and S4) than the intact-side thresholds (Figure 1). Thus, for the more proximal biceps muscle, the two subjects with an in utero amputation (S3 and S4) had an asymmetric threshold pattern similar to that observed after traumatic amputation, while the two agenesics showed symmetric thresholds.

**Figure 2:**
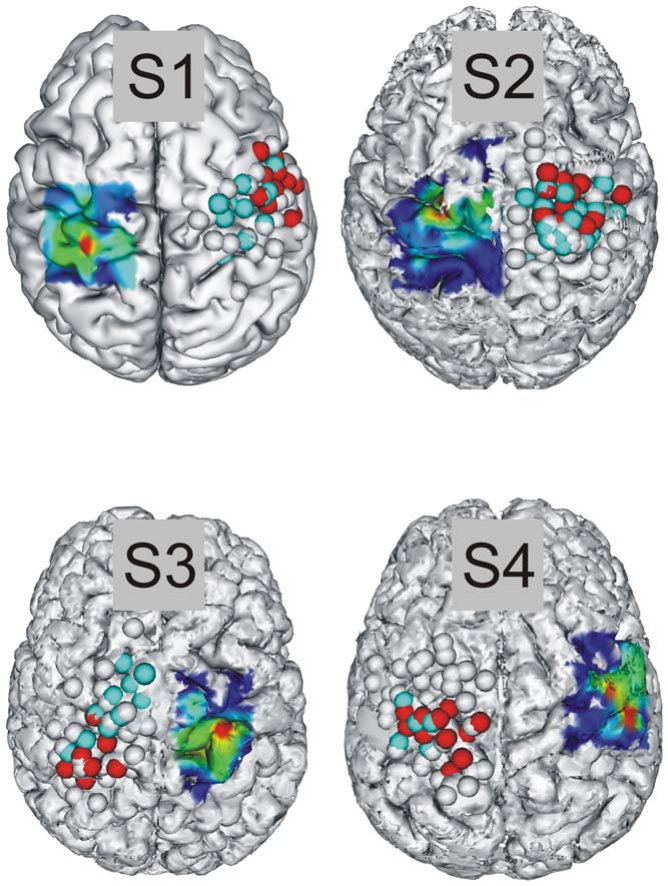
Relationship between the map of an intrinsic hand muscle (FDI) and points stimulated on the hemisphere contralateral to the missing limb. For all four subjects the points stimulated on the hemisphere contralateral to the missing limb largely covered the “probable” hand area in this hemisphere. Sites at which stimulation produced MEPs of at least 10% of the maximum average MEP amplitude are marked in blue for the biceps, red for the FDS, and grey points represent sites that were stimulated but did not evoke MEPs in either biceps or FDS. On the interpolated maps the transition between dark and light blue corresponds to approximately 10% of the maximum average MEP amplitude. Table 2 shows the percentage of Maximum Stimulator Output used to construct each map.

Figure 2 shows the anatomical MRIs of each of the four subjects showing the points that were stimulated on the hemisphere contralateral to the missing limb as well as the map of the intact FDI. Because FDI is an intrinsic hand muscle it is a good indicator of the location of the hand area in the motor cortex. By mirroring this muscle map onto the hemisphere contralateral to the missing limb we can identify the homologue of the “presumed” hand area and thereby determine whether TMS in the region most likely to contain the hand's representation provoked phantom sensations. For the hemisphere contralateral to the missing limb, sites at which stimulation produced average MEP amplitudes of at least 10% of the maximum average MEP amplitude (taken from the site where the average of the six MEPs was greatest) are marked in blue for the biceps, red for the FDS, and grey points represent sites that were stimulated when making either the FDS or Biceps maps but did not evoke MEPs in the muscle being mapped. For most subjects illustrated in Figure 2, a large number of sites stimulated when making the biceps map also evoked MEPs in FDS and vice versa. Thus, in red we show those sites that evoked a minimum MEP amplitude in Biceps when mapping at biceps-appropriate parameters and likewise for FDS (blue points). The degree of overlap between the two muscle representations does not seems to be related to the cause of the amelia, as in subjects 2 and 4 the two muscle representations are highly overlapping (shown by the intermingling of blue and red points), while for S1 and S3 the muscles have relatively separate representations. When compared with the same type of map for the intact sides (data not shown) there was no systematic difference in the degree of overlap between the biceps and FDS muscles on the intact and missing limb sides.

**Figure 3:**
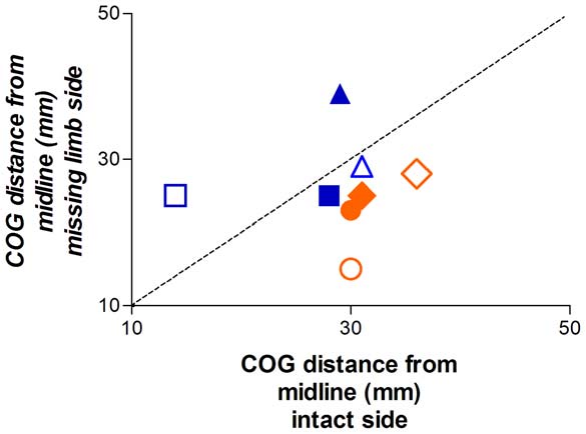
Distance of Centre of Gravity for BB (open symbols) and FDS (filled symbols) maps from the midline on the missing limb and intact sides of each subject (blue = agenesics; orange = in utero amputees). In three of the four subjects, the CoG of the stump muscle FDS was more medial on the missing limb side than the intact side. Similarly, three of the four subjects had a BB representation that was more medial on the missing limb side than the intact side (triangle = S1, square = S2; circle = S3, diamond = S4).

Figure 3 shows the distance from the midline for the biceps and FDS map CoGs on the missing and intact limb sides. For 6 of the 8 comparisons the muscle's CoG was more lateral on the intact than the missing limb side of the body. Interestingly, the two cases for which the muscle CoG was more lateral on the missing limb side were from subjects 1 and 2, both of whom were missing their hand as a result of agenesis rather than in utero amputation. Overall, regardless of the cause of the amelia, we observed the opposite of what is generally assumed to occur after traumatic limb amputation in which the missing limb muscles are represented more laterally than the homologous intact limb muscles. It should be noted, however, that recent evidence challenges this assumption by showing that TMS muscle representation maps in the amputated limb of above-elbow traumatic amputees do not always shift laterally [33].

**Figure 4:**
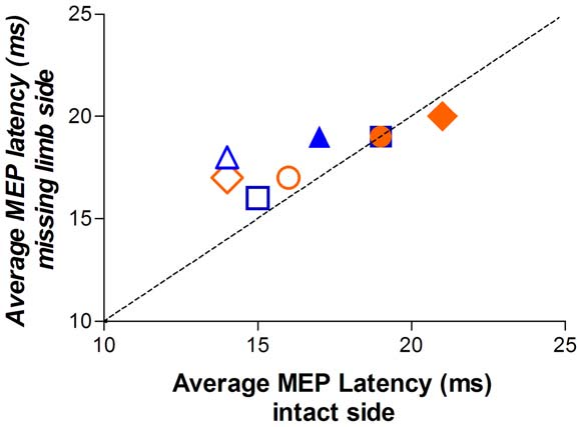
Average MEP latency for BB (open symbols) and FDS (closed symbols) for the missing limb and intact sides of each subject (blue = agenesics; orange = in utero amputees). There were no systematic latency differences between the two sides of the body, despite the absence of the majority of the forearm and all of the hand muscles on the side with the missing hand. Note that the subject represented by the square and the subject represented by the circle both had latencies of 19 ms on the intact and amputated sides – the overlapping of these two points explains why the FDS latencies for the square are not visible (triangle = S1, square = S2; circle = S3, diamond = S4).

Figure 4 shows the average MEP latency for the biceps and FDS muscles on the intact and missing limb sides. In three of the four subjects MEP latencies were slightly shorter for the biceps on the missing limb side than the intact side. For the FDS they were identical in two subjects and slightly longer on the missing limb side in the other two. Thus, there were no systematic differences in MEP latencies between the two sides of the body.

In order to assess whether there were any systematic differences between TMS parameters for the two types of subjects (agenesics (S1 and S2) versus in utero amputees (S3 and S4)) we also calculated the map area for all five muscles mapped in each of the four subjects (Table 3). For biceps and FDS the average map area for intact limb muscles was almost equal for the two subject types, whereas for muscles on the missing limb side map area was more than 40% larger in the agenesics. The FDI map area (intact side) also tended to be larger in agenesics than in utero amputees.

**Table 3:** Map area (number of stimulated sites with a mean MEP amplitude ≥0.05 mV).

Subject	Muscle	Map Area	
		Missing Limb	Intact Limb
S1	BB	13	8
	FDS	15	15
	FDI	-	19
S2	BB	22	15
	FDS	21	11
	FDI	-	16
S3	BB	11	5
	FDS	10	13
	FDI	-	13
S4	BB	6	12
	FDS	22	22
	FDI	-	13

## Discussion

We studied four subjects with congenital absence of a limb and no phantom sensations and found that TMS applied over the motor cortex contralateral to the missing limb produces contractions in stump muscles but does not evoke phantom movement sensations. Cohen and colleagues [17] applied TMS to the motor cortex of a single subject with congenital absence of the hand and no phantom limb sensations and reported a similar result. In contrast, in an individual with congenital absence of both hands but extremely vivid phantom sensations Brugger and colleagues [11] reported that TMS over the intact deltoid muscle representation evoked the sensation of movement in the phantom hand. At first glance this appears to closely resemble the findings reported after TMS over the sensorimotor cortex of traumatic amputees with vivid phantom limbs [19]. Indeed, similar to traumatic amputees, Brugger's patient provided precise descriptions of the stimulation-induced phantom movements. Unlike traumatic amputees, however, this patient never reported twitch-like movements in the phantom and often felt the phantom move a long time after the stimulation. These differences are very important, as they suggest that the sensation of movement experienced by Brugger's patient might arise from activation of a different type of representation from that which gives rise to TMS-induced phantom movement sensations in traumatic amputees. This, together with our finding that TMS over the motor cortex of upper limb amelic subjects without a phantom limb does not evoke phantom sensations, suggests that in upper-limb amelics a hand movement representation either does not develop within the motor cortex or it matures differently from those representations that exist in healthy controls and traumatic amputees.

One interpretation of our results is that amelics who do not spontaneously report the presence of a phantom limb might not possess an intact body schema, or at least not the same type of body schema as that possessed by traumatic amputees. Support for this idea comes from a behavioural study by Nico and colleagues [43] who performed a left/right hand judgement task with 16 traumatic amputees and three individuals born without a limb. The task required subjects to mentally simulate rotation of images of hands in various positions in order to judge the laterality of the hand. They found traumatic amputees responded less accurately and slower than controls when the image was of their missing hand. In contrast, subjects born without a limb showed a level of performance similar to that of the control subjects, with one interesting exception; unlike controls, when the image observed was in an unnatural posture subjects born without a limb did not have longer reaction times when the judgement concerned their missing hand. The authors interpreted this as evidence that the mental simulation performed by the amelic subjects was not affected by physical constraints of the limb. In other words, it is unlikely that they made reference to their body schema to perform the task, perhaps because their body schema does not contain information about the missing limb.

In addition to examining whether motor cortex stimulation could evoke phantom movement sensations we also wanted to know whether the organisation of the motor cortices of these four subjects was analogous to that observed in traumatic upper-limb amputees. We found no evidence for a large between-hemisphere asymmetry in the location of the arm representation, reflected by the near-symmetric locations of the biceps and forearm flexor CoGs in both hemispheres. At first glance it is surprising that the absence of part of the forearm and the hand since birth results in no major asymmetry in the location of the arm region of the motor cortex as measured by muscle CoGs, but this is consistent with other studies. The congenital amputee studied by Brugger and colleagues had no elbows or forearms, but her deltoid CoGs fell within the range observed in normal subjects [11]. Furthermore, TMS studies of traumatic upper-limb amputees do not always show shifts in the location of arm muscle representations [33], [44], and when a shift is present the stump muscle representation can be either more lateral than the homologous muscle's representation [25], [29], [31], or more medial [30]. The degree of symmetry in the location and size of muscle maps in normal subjects also appears to be variable. Studies investigating upper-limb muscle representations either report no between-hemisphere asymmetries [34], [45], [46], or an asymmetry that is related [47] or unrelated [48] to the subject's handedness. The absence of a systematic shift in muscle locations after amputation and the finding of a symmetry in some normal subject studies and an asymmetry in others, might be due to the fact that the motor representations of proximal and distal upper-limb muscles overlap substantially [49], [50] and do not show an orderly somatotopy similar to the one that exists between major body segments like the face, upper-limb, and lower-limb [51].

While the location of the arm representation (measured from the biceps and FDS CoGs) was relatively symmetric across the two hemispheres, we observed a clear difference in muscle activation thresholds between the FDS on the missing limb and intact sides. Thresholds were always **higher** by an average of 12% MSO for the stump muscle (FDS on the missing limb side). It is important to note that while a threshold asymmetry is also observed after traumatic amputation, the direction of this difference is inversed; in above-elbow traumatic amputees stump muscle thresholds are **lower** (range 10 to 17% MSO) than thresholds for the homologous muscle on the intact side [e.g. 17], [19], [25], [30], [33], [41]. In contrast, thresholds for upper-limb muscles in normal subjects are very symmetric [35], [45], [47], [52], [53], [54], [55], [56], and the few studies that do find differences report values smaller than those observed in either traumatic amputees or in our amelic subjects [e.g. 57], [58].

While the threshold data for FDS reveal a consistent asymmetry across all four subjects, the biceps thresholds show a different pattern depending upon the cause of the amelia. The two subjects with agenesis had symmetric biceps thresholds whereas the in utero amputees showed a pattern similar to that observed after traumatic amputation, with lower thresholds on the missing limb side. Lower stump muscle thresholds after traumatic amputation are thought to be due to disinhibition of the motor cortex induced by the large scale deafferentation that follows amputation. This disinhibition might permit the “reappearance” of the otherwise latent representation of the missing hand. In both agenesics and in utero amputees we not only failed to observe lower (or even equal) motor thresholds for stump muscles, we found instead that stump muscles had *higher* thresholds. This might be a sign that the motor cortex contralateral to the missing limb receives strong inhibitory inputs which, if there were a latent representation of the missing limb, might prevent its activation by TMS. Interestingly, if there are strong inhibitory inputs acting on the motor cortex contralateral to the missing limb, these inputs act selectively on muscles within the stump, as the more proximal biceps representation showed no asymmetry in the two agenesic subjects whereas in the other two subjects it appears to have undergone a disinhibition similar to that observed after traumatic amputation.

### Conclusion

The motor cortex of traumatic amputees who report the presence of a phantom limb contains representations of movements of the amputated limb which can be activated by stimulating the motor cortex [19]. Here we show that this is not the case for individuals with congenital absence of a limb who do not report the presence of a phantom limb. We interpret this as evidence that their motor cortex does not contain a representation of the missing limb, or that if such a representation exists it receives extremely strong inhibitory inputs and cannot be accessed either voluntarily or following intense external stimulation. Can these results be extended further and taken as evidence that amelics do not possess a body schema with four limbs?

The fact that our sample did not include any amelics with a phantom limb does not necessarily lead to the conclusion that all amelics lack a sensorimotor representation of their missing limb, especially given the finding that TMS over the motor cortex of a bilateral upper-limb amelic evoked phantom limb movements [11]. As stated before, however, there are clear differences between TMS-induced sensations in the amelic with phantom limbs and in traumatic amputees with phantom limbs. In traumatic amputees TMS evokes twitch-like sensations in the phantom that immediately follow the stimulation and resemble the twitches evoked in the intact limb [19]. In contrast, the patient studied by Brugger and colleagues never reported twitches in the phantom, often felt the phantom move one or more seconds after the stimulation, and reported phantom movements at sites that did not produce MEPs. Since the latency and “motor quality” of this patient's phantom movements do not resemble those reported by traumatic amputees they probably arise from indirect activation of regions beyond the primary motor cortex rather than via direct activation of a representation of the missing limb within the motor cortex. Indeed, stimulation-induced activity could spread to other brain areas which contain some type of sensorimotor representation of the missing limb. This, together with the current results, suggests that amelics (including those who report phantom sensations) do not possess a motor representation of the missing limb within the motor cortex similar to that found in traumatic amputees. Despite this, some form of sensorimotor representation of the limb might exist in other brain regions. Indeed, the body schema cannot be localised to a single brain area, but arises from the dynamic exchange of information within a network containing multiple brain areas, including the posterior parietal, premotor, and primary sensorimotor cortices as well as other subcortial and spinal circuits [59], [60]. Thus, the absence of a representation of the missing limb in the motor cortex does not exclude the possibility that amelics possess a body schema that includes some form of sensorimotor representation of all four limbs.

We recently proposed that two levels of hand movement representation exist within M1: on one level limb movements are specified in terms of arm and hand motor commands, and on another they are specified as muscles synergies [23]. After traumatic amputation reorganisation appears to take place at the muscular map level, leaving intact the motor commands capable of signalling movements of the phantom limb such as those experienced during voluntary phantom limb movements [20] or following TMS stimulation of the motor cortex [19]. One could speculate that when a limb never develops or amputation occurs in utero, neither level of motor representation develops within M1. This is probably because in the absence of motor experience, representations of movement synergies cannot be processed. Within this framework, phantom movement sensations reported by a few upper limb amelics are probably not related to a genuine sensorimotor representation of the missing limb, but might instead be driven by vision and therefore mediated by other cortical areas such as the parietal cortex, a region known to be involved in the visuo-motor representation of complex hand movements [61]. In future studies it will be important to characterise the exact nature of phantom sensations in congenital amputees, whether these are related to the organisation of the sensorimotor cortex, and whether the absence of a phantom limb is a valid criterion for saying that the body schema does not contain a representation of the missing limb.
